# Use of Physical Accessibility Modelling in Diagnostic Network Optimization: A Review

**DOI:** 10.3390/diagnostics12010103

**Published:** 2022-01-04

**Authors:** Camille Chênes, Heidi Albert, Kekeletso Kao, Nicolas Ray

**Affiliations:** 1 Institute for Environmental Sciences, University of Geneva, 1205 Geneva, Switzerland; camille.chenes@unige.ch; 2 FIND, Cape Town 7925, South Africa; heidi.albert@finddx.org; 3 FIND, 1202 Geneva, Switzerland; kekeletso.kao@finddx.org; 4 GeoHealth Group, Institute of Global Health, University of Geneva, 1202 Geneva, Switzerland

**Keywords:** diagnostic network optimization, physical accessibility, referral system

## Abstract

Diagnostic networks are complex systems that include both laboratory-tested and community-based diagnostics, as well as a specimen referral system that links health tiers. Since diagnostics are the first step before accessing appropriate care, diagnostic network optimization (DNO) is crucial to improving the overall healthcare system. The aim of our review was to understand whether the field of DNO, and especially route optimization, has benefited from the recent advances in geospatial modeling, and notably physical accessibility modeling, that have been used in numerous health systems assessment and strengthening studies. All publications published in English between the journal’s inception and 12 August 2021 that dealt with DNO, geographical accessibility and optimization, were systematically searched for in Web of Science and PubMed, this search was complemented by a snowball search. Studies from any country were considered. Seven relevant publications were selected and charted, with a variety of geospatial approaches used for optimization. This paucity of publications calls for exploring the linkage of DNO procedures with realistic accessibility modeling framework. The potential benefits could be notably better-informed travel times of either the specimens or population, better estimates of the demand for diagnostics through realistic population catchments, and innovative ways of considering disease epidemiology to inform DNO.

## 1. Introduction

The importance of attaining Universal Health Coverage, a key target of the 2030 Sustainable Development Goals no. 3, is no longer contested [1]. Countries commit to ensuring access to affordable and quality healthcare services. A major component in achieving this goal is to consider access to diagnostic services. Indeed, essential diagnostics are the first step towards accessing appropriate care, influencing 70% of health decisions [2]. By prescribing the appropriate treatment, diagnostics help reduce excessive drug use, thereby reducing the emergence of antimicrobial resistance and also minimizing financial losses. In addition, they are essential for the early detection of diseases, enabling the monitoring and control of the spread and emergence of infectious diseases [2,3]. A recent study [4] on 10 low- and middle-income countries (LMICs) showed that major gaps in diagnostic availability exist in some of these countries, particularly at the primary care level. Another recent publication on nine LMICs and two states in the USA shows that access to laboratory tests other than malaria and HIV are relatively low, with only 10–20% of the population covered [5].

To obtain a comprehensive view of their availability, access, and use, diagnostics must be placed in their overall context. Diagnostic networks encompass all the components, considering not only tests performed within laboratories, but also those conducted by devices located in communities or clinics, outside the laboratory setting, as well as the specimen referral system, which links referral and reference sites [6]. Initially based on manual methods and expert consensus, the design and planning of diagnostic networks has recently moved towards a greater use of advanced digital tools and data analytics, notably using models link to Geographic Information Systems (GIS), targeting “diagnostic network optimization” (DNO, see [6] and references therein). DNO usually implies mathematically solving the best combination of variables to optimize outputs such as costs, capacity, turnaround time of results and device placements. A sub-branch of DNO, route optimization (RO), also aims to highlight the best routes for specimen referral. The ultimate goal of DNO is usually to maximize accessibility to testing centers for the population, while minimizing overall costs, subject to country-specific constraints and assumptions applied. [6,7,8].

Consideration of the infrastructure that facilitates the transport of specimens (e.g., roads), obstacles that impede this transport (e.g., rivers, forests), and also transport modes and speeds that provide information on how specimens are transported between sites can make route optimization and other accessibility models more realistic, especially in LMICs [9,10]. Although these analyses typically do not include costs, accurate transport modes and speeds determined by experts in the field provide better estimates of parameters for accessibility models [9,10]. Such approaches have also been used to model population accessibility to health services, such as primary healthcare [11], emergency obstetric and neonatal care [12,13], and community health services [14]. Although this dimension is not currently part of the DNO, it may provide an interesting additional functionality that could be added in the future. For this reason, population accessibility has been considered in this review.

Given the recent emergence of DNO and the recent advances in applying accessibility modelling in the framework of numerous other health services, the aim of our scoping review is to determine if and how physical accessibility has been used to inform the optimization of diagnostic networks. We only consider diseases that can be identified with a diagnostic device, requiring a specimen to be collected, enabling an overall review of diagnostic networks.

## 2. Materials and Methods

We followed the scoping review methodology recommended by Arksey and O’Malley [15], and we adhered to the 2020 PRISMA guidelines [16].

### 2.1. Eligibility Criteria

All publications involving assessment or optimization of diagnostics networks through the use of distance- or time-based metrics, or using concepts linked to physical accessibility to the network or population accessibility, were included in the selection process. We targeted journal articles, book chapters, short communications, or presentations written in English. Studies taking place either in high-income or low- and middle-income countries were kept, although at the end of the selection process, these two sets were treated distinctly. All studies that could not be related to at least one diagnostic or that did not use geospatial analyses or optimization strategies were excluded.

### 2.2. Search Strategy

As we targeted studies involving geographical accessibility and diagnostic network optimization, we first identified four keyword groups: (1) Accessibility, (2) Geographical, (3) Diagnostics, and (4) Optimization. A preliminary literature review, as well as a discussion among co-authors, allowed the identification of keywords within each category. Thus, the keywords *access*, *travel time*, *distance* and *transport time* have been used to describe accessibility. To ensure that emerging studies have a spatial dimension and to consider only physical accessibility, the keywords *geographic*, *geospatial*, *spatial*, *geographic information system,* and *GIS* were used. For diagnostics, it was particularly important to capture the associated network dimension in order to avoid retaining numerous purely medical publications. Specimen transport, an important factor inherent to diagnostic networks, was also emphasized by using the keywords *diagnostic network*, *diagnostic service*, *diagnostic system*, *sample transport network*, *sample transport system*, *route optimization*, *hub and spoke,* and *turnaround time*. Finally, to ensure that the publications contain an optimization effort, the keywords *optimize*, *improve*, *design*, *maximize,* and *cost-efficiency* were used in the search strategy. The final detailed search queries are found in Supplementary Material File S1. The search was launched in PubMed and Web of Science on 21 May 2021. New publications were verified until 12 August 2021. Following the database search, manual and snowball searches were conducted, adding publications to the selection process.

### 2.3. Publication Selection

All publications resulting from the search process were exported to Microsoft Excel version 2016 [17]. Duplicates were removed. Two co-authors (NR and CC) screened the titles and abstracts of the articles and independently selected those that met the eligibility criteria. Results were compared and disagreements discussed among the co-authors. If a consensus was not found, the article was selected for full text reading.

### 2.4. Charting the Data

All selected publications were fully read by CC, and all-important pieces of information summarized in a table containing the following fields: authors, journal, study design, country, subnational region, geographical aggregation, time period, diseases concerned, diagnostic type, tier level or referral, optimization solution, accessibility measures, and units (Table S1).

## 3. Results

### 3.1. Overview of the Literature Search

**Figure 1:**
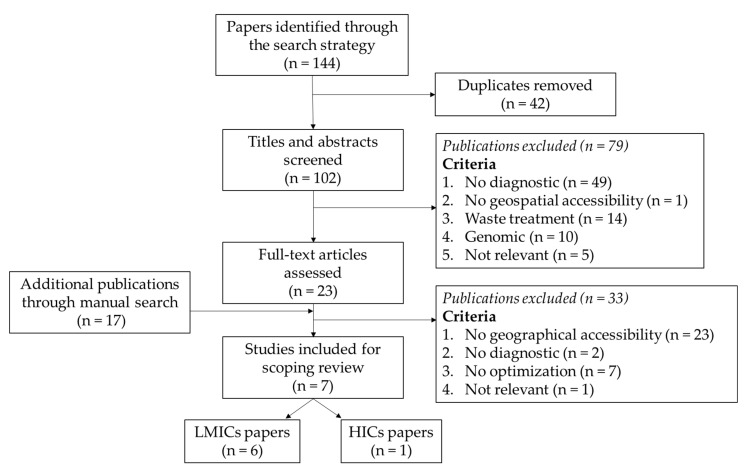
Overview of the article selection process and selected article at each step.

The search process resulted in the identification of 136 articles. A total of 35 duplicates were removed. After the evaluation of the titles and abstracts, 23 publications were retained. Many publications were related to the optimization of waste treatment (n = 14) or focused only on the genomic aspects of diagnostics (n = 10) and were discarded. At this stage, 17 other publications, from manual and snowball searches, were added. A total of 40 publications were fully read. Seven research papers were finally included (Figure 1). Publication dates for these articles range from 2014 to 2021.

### 3.2. Disease and Associated Diagnostics

Two publications addressed Tuberculosis, both considering GeneXpert diagnostic devices. Three publications dealt with HIV, with one study examining disease detection with CD4 diagnostic tools, and two studies focusing on antiretroviral therapy and viral load. Another publication dealt with lower respiratory tract infections, by testing C-reactive protein. The latest publication focuses on Onchocerciasis, a neglected tropical disease.

### 3.3. Geographical Patterns of Studies

The studies targeted seven different countries including Ghana [18], Zambia [8,19], Lesotho [20], South Africa [21], Democratic Republic of the Congo, and Angola [22]. Finally, the only study based on a high-income country was conducted in the UK. Four of these studies were conducted at national level [8,19,20,21].

### 3.4. Accessibility Measures

Three types of geographic accessibility measures were identified: those using travel time [8,19], those using distance [20,21,22,23], and one combining these two measures [18]. The studies used different strategies to measure physical accessibility: using ArcGIS Costdistance function (ESRI, Redlands, CA, USA), for a unique transport mode and a mean travel speed [18]; using ArcGIS ModelBuilder to solve a Vehicle Routing Problem, considering several transport modes and speeds [19], or only a single transport mode [8]; using the Open-Source Routing Machine to compute walking distance [23]; using distance data along transport routes where known, otherwise using a distance adjustment factor to define them [20,22]; and finally, using Euclidean distances to determine the coverage of a health center [21].

### 3.5. Referral System

Only one study does not consider referrals at all but focuses on population accessibility to health care services [18]. Regarding the referral system, the majority of studies are concerned with specimen referrals [8,19,20,21,22], while one study considers patient travel [24]. For studies looking at specimen referrals, one includes all levels of health centers in their analysis [20], others link specimens from all types of health facilities to centralized laboratories [19,21], one focuses on the transport of specimens between Point-of-Care and centralized laboratories [8], while the latter is concerned with referrals from community centers to general hospitals [22]. The publication involving patient travel implies patient reference from their usual General Practitioner to another GP location or to pharmacies [23].

Several publications propose a motorbike as a mode of transport [8,18,20]. Two studies include several modes of transport [19,22], one only includes walking [23], and the last one does not include any specified mode of transport [21].

### 3.6. Optimization Goal

In over half of the studies, accessibility measures are used as an input for cost calculations [8,19,20,22]. These publications optimize their diagnostic networks by proposing various scenarios, based on geospatial models, and choosing the one that maximizes coverage and minimizes costs (transport, or overall costs). Another study models an integrated tiered service delivery that aims to improve the coverage of CD4 testing services, reducing turnaround time and enabling tests cost savings [21]. Other publications use accessibility directly for their network optimization, targeting new health center locations, with one of the studies using the ArcGIS Location-Allocation function [23] and the other using MapInfo and an SQL search [18].

### 3.7. Data Used

Several publications used a road layer, sourced either from national entities [18] or from OpenStreetMap [23,24], or by assembling a road network [8,19,20,22]. In the last publication [21], no specific information on road networks was used. To determine accessibility, studies also used population distribution data [18] or centroids of densely-populated areas [23]. Others used the test demand covered by each facility, based on historical or current test volumes, workload, or facility capacity [8,19,20,21,22]. Finally, the health centers and laboratories data were collected from previous surveys [8,18,19,21], from national program registration [20,22], or from open data [23]. The main pieces of information from the charting process are summarized in Table 1.

## 4. Discussion

This scoping review identified only seven publications that attempted to enhance diagnostic networks using physical accessibility. The paucity of publications may be related to the fact that the DNO initially originated from the commercial sector, where publication is not a driver and that the DNO field is recent in the public health. Six studies were conducted in low- and middle-income African countries. These studies have mainly the same optimization objectives, i.e., that health centers and their referral systems cover the maximum percentage of the population while minimizing costs (transport, tests, or overall costs), or simply that the population travel time to a health center with diagnostic capacity is reduced. However, the strategies to achieve these objectives differ among studies, except for two studies that are from the same team, and thus use the same data and geospatial model.

Studies from LMICs focus on the optimization of diagnostic networks for tuberculosis, HIV and Neglected Tropical Diseases. The only study from a high-income country focuses on lower respiratory tract infections. Publications from high-income countries were mostly excluded from the scoping review due to the health diagnostics addressed. Most of them focused on cancers, mental illnesses, strokes, or rare air pollution-related diseases, and thus did not meet the criteria for the diagnostics covered by this review. For LMICs, a large number of studies have been undertaken on the accessibility of health care facilities, but without focusing on specific diagnostics. Otherwise, although diagnostics have been analyzed, no optimization or spatial accessibility has been proposed.

Throughout our literature search, we found the Hub-and-Spoke model was often mentioned and used to improve the health network for high-income countries. This model consists of a health infrastructure, called a hub, offering a wide range of services and advanced diagnostic tools, which is connected to a multitude of secondary health facilities, offering basic services, called spokes. These networks have the advantage of being easily adaptable, by adding spokes or identifying other hubs, and are effective and efficient as they provide quick access to healthcare services for patients, while minimizing costs [25]. For example, a study conducted in the Northern Territory of Australia optimized the location of hubs, using the ArcGIS location-allocation function, and showed that the total mean travel time of the population to access a hub service could be reduced from 25 to 19 min [26].

For LMICs, the Hub-and-Spoke model is mentioned in only four of the 40 assessed publications but they were not selected because no optimization or accessibility measures were proposed [27,28,29,30]. However, the implementation of this model has been successfully conducted in some countries [28,30]. For instance, the introduction of a centralized strategy for Early-Infant HIV Diagnostic services has led to a reduction in the turnaround time of 46.9% and a reduction in costs of 62% [27,30]. In contrast, some countries, such as Haiti and Ethiopia, have opted for a decentralized model for their specimen referral network, which has also led to increased access to testing facilities and reduced turnaround times [27,29,31]. Similarly, Glencross et al. [21] have also proposed a decentralized model to optimize the delivery service, reaching the most remote locations.

Another method used to improve access to health centers is the use of telemedicine. This approach does not logically include accessibility measures, as it is mainly based on remote consultations, and therefore no publications dealing with this aspect have been retained. However, telemedicine offers advantages and, being mainly used in mental health and recovering therapies in high-income countries [32,33,34,35], it also increases equity of access to care for the population in LMICs. Using asynchronous teleconsultations to overcome poor internet access in some remote areas [36], telemedicine can provide consultations with specialists, reduce missed working days, and enhance the referral system offering early detection and referrals to a suitable diagnosis, thus reducing costs. At the same time, the increase of mobile phones and the creation of applications have helped to reduce the turnaround time, by sending the diagnostic result directly to the patient’s phone, thus eliminating the need for a return journey [37].

In general, the geospatial data used to assess physical accessibility are either incomplete, simplified, or non-existent. Some studies define a distance radius to establish turnaround times [21,29,30] or modelled the road network by connecting nodes and lanes from existing information [20,38]. Even models using a road layer simplified the analyses by using an average speed or a single transport mode [18,20,26]. To improve the accuracy of the results, geospatial data provided by OpenStreetMap can be a good alternative when official data do not exist or are not readily available [23]. However, maps and data from cadastral or online sources can provide a better overview of transportation networks and barriers to movement, and therefore can offer more accurate results. Ideally, expert information allows one to obtain data that better reflect the reality in the field. It can provide information on the effect of seasonality on travel time, or on travel time specific to a target population, such as children [39] or pregnant women [13]. As a measure of overall access to diagnostics, our seven selected studies either made use of information on test demand or historical or current volume [8,19,20,21], or factored in population distribution or centroids of densely-populated areas [18,23]. Using the population distribution and simple travel-time catchment around health facilities, access and population coverage of the network were computed [18]. In [23], the centroids and their associated population were used as starting points to estimate the distance to the nearest care center and the demand, respectively. Incidence data were used in three publications to estimate demand. However, most of the selected studies did not use epidemiological incidence or prevalence data to get a better sense of the potential demand for testing, as pre-existing data on test volumes or population densities are not always the best proxies for projecting future demand.

### 4.1. Limitations of the Study

This scoping review on the use of physical accessibility in the optimization of diagnostic networks presents several limitations. Firstly, only publications written in English were screened. Secondly, only the PubMed and Web of Science databases were used in the selection process, potentially hindering access to articles from other sources. Finally, although we used a large number of relevant search keywords decided on the basis of an initial literature review and the experience of the co-authors, we may have overlooked a keyword or missed some studies. This was hopefully mitigated by our secondary snowball and manual searches.

### 4.2. Conclusions and Future Research

Our review of the literature indicates that very few published studies targeting DNO make use of accessibility models informed by field- or expert-based information on speed and mode of transport of either samples or patients. This may impact on the realism of the model results and is at odds with the growing literature that uses this type of modeling approach for various other health services [10,40,41,42]. This seemingly sub-optimal use of the recent physical accessibility models may come from the recent consideration of the importance of data analytics in DNO [6]. It may also reflect a decoupling of the types of geospatial tools traditionally used for optimizing supply chains (of diagnostics in this case but it originates from the commercial sector), with the category of tools that inform health systems strengthening and scaling up.

These gaps encourage several lines of research that could benefit the broad field of DNO, and especially RO and its applications in LMICs. First, realistic ways of estimating the travel times of samples within a diagnostic network could benefit from the studies that have elicited and used national or regional expertise on the modes and speeds of transport to optimize health systems. This information can supplement (or complement) the database from tracked vehicles that are seldomly available in most countries. Second, similar approaches have been used in many countries to estimate the modes and speeds of the transport used by the population travelling to a health service. This information could be used by adjusting it for the target population linked to the diagnostics of interest. Coupled with recent high-resolution population distribution maps (e.g., data sets from worldpop.org, [43]), estimated population catchments around points of care can be modelled, which could provide better information about estimates of the demand for diagnostics. Third, epidemiological data on disease prevalence could be more widely used to provide better information about the demand for diagnostic. A DNO could potentially give different results if the demand side is modelled based on population alone or the epidemiology of the target disease. A recent study using malaria incidence in Niger demonstrated that this is the case when modelling the deployments of community health workers [14]. To summarize, physical accessibility modeling can improve RO by providing accurate information on travel scenarios and times, as well as on barriers to movement. Furthermore, population accessibility to health care services can directly inform DNO by providing information on the potential demand, which can be coupled with epidemiological data.

Finally, the future availability of easier-to-use DNO software may help democratize their usage and their coupling with other geospatial approaches for health system optimization. One promising avenue is the pilot project OptiDX [44,45,46], which is an online and easy-to use DNO tool scheduled to be made freely available to African countries in 2022 to help guide investment decisions. Coupled with open-source accessibility modelling tools such as AccessMod [9], there is a large potential for improving the results of DNO models. This would translate into better-informed decisions for health system strengthening, moving towards Universal Health Coverage.

## Figures and Tables

**Table 1:** Summary of information from the selected articles.

Ref.	Country	Disease	Accessibility Model	Referral System	Diagnostic Network Optimization Strategies
[18]	Ghana (sub-national)	TB	Distance: along the road network; Travel time: Costdistance, average 20 km/h motorized tricycle	-	51 additional TB testing health facilities located <10 km from population: MapInfo and SQL query. Only study interested in population accessibility.
[20]	Lesotho (national)	TB	Travel distance: along the road network or using a distance adjustment factor	Sample	Diagnostic network scenarios modelled using Supply Chain Guru software. The optimized scenario is the lowest overall cost solution that meets all constraints.
[8]	Zambia (national)	HIV VL	Travel time: ArcGIS Network Analyst tool, Salesman Problem	Sample	ArcGIS Location-Allocation function, maximizing ART POC facilities coverage and Geospatial model that minimizes driving time and minimizes overall costs.
[19]	Zambia (national)	HIV VL	Travel time: ArcGIS Network Analyst tool, Salesman Problem	Sample	ArcGIS Location-Allocation function, and geospatial model that maximized the Sample Transport Network, while minimizing the transport cost. Two sample transportation scenarios: district-bounded and borderless scenarios.
[21]	South Africa (national)	HIV	Distance: Euclidean distance (<100 km)	Sample	Integrated tiered service delivery model that ensure CD4 testing are accessible at health facilities within 24–48 h local turnaround time and contain test costs.
[23]	UK (sub-national)	Lower Respiratory Tract Infections	Distance: along the road network, Open-Source Routing Machine	Population	ArcGIS Location-Allocation function. Mathematical model allocates C-reactive protein testing location to minimize the overall travel and ensuring that patients never have to travel more than a predefined maximum distance.
[22]	DRC and Angola (sub-national)	Onchocerciasis	Travel distance: along the road network or using a distance adjustment factor	Sample	DNO can help evaluate alternate sampling strategies to bring opportunities for overall cost savings.

## Supplementary Materials

The following supporting information can be downloaded at: https://www.mdpi.com/article/10.3390/diagnostics12010103/s1, File S1: Database search terms; Table S1: Complete charting Table.
