# Supplementary material for: Use of Physical Accessibility Modelling in Diagnostic Network Optimization: A Review
Source: Diagnostics (Basel). 2022 Jan 4;12(1):103. doi: 10.3390/diagnostics12010103 (PMC8774366; doi:10.3390/diagnostics12010103)
Supplement: Supplementary file 1 [file diagnostics-12-00103-s001.zip › diagnostics-1527697-supplementary.pdf]

File S1. Database search terms.
Query formulas developed for PubMed as well as Web of Science.

PubMed search terms:
("access*"[Title/Abstract] OR "travel time"[Title/Abstract] OR "distance*"[Title/Abstract] OR "transport time"[Title/Abstract]) AND ("geographic*"[Title/Abstract] OR "geospatial"[Title/Abstract] OR "spatial"[Title/Abstract] OR "GIS"[Title/Abstract] OR "geographical information system"[Title/Abstract]) AND ("optimiz*"[Title/Abstract] OR "improv*"[Title/Abstract] OR "design*"[Title/Abstract] OR "maximiz*"[Title/Abstract] OR "cost efficiency"[Title/Abstract]) AND ("diagnostic network"[Title/Abstract] OR "diagnostic networks"[Title/Abstract] OR "diagnostic service"[Title/Abstract] OR "diagnostic services"[Title/Abstract] OR "diagnostic system"[Title/Abstract] OR "diagnostic systems"[Title/Abstract] OR "laboratory network"[Title/Abstract] OR "laboratory networks"[Title/Abstract] OR "testing facilit*"[Title/Abstract] OR "screening facilit*"[Title/Abstract] OR "specimen referral system"[Title/Abstract] OR "sample transport system"[Title/Abstract] OR "route optimization"[Title/Abstract] OR "hub and spoke"[Title/Abstract] OR "turnaround time"[Title/Abstract])

Web of Science search terms:
TS=((access* OR "travel time" OR distance OR "transport time") AND (geographic* OR geospatial OR spatial OR GIS OR "geo*information system") AND ("diagnostic network*" OR "diagnostic service*" OR "diagnostic system*" OR "laboratory network*" OR "testing facilit*" OR "screening facilit*" OR “specimen referral system” OR “sample transport system” OR “route optimization” OR “hub and spoke” OR “turnaround time”) AND (optimiz* OR improv* OR design* OR maximiz* OR “cost efficiency”))

Table S1. Complete charting Table.

A summary of the information obtained from the 40 publications selected for further reading. The 7 publications selected for the final review are highlighted in blue.

N°	Source	Title	Authors	Journal	Study design	Country	Subnational region	Geographical aggregation	Time period	Diseases	Diagnostic type	Health tier / Referral	Optimization tools/solutions	Access. variable	Access. unit
1	Database search	Geographical accessibility to public health facilities providing tuberculosis testing services at POC in the upper east region, Ghana	Kuupiel et al. (2019)	BMC Public Health	Geospatial modelling	**Ghana**	Upper East Region	13 districts	2018-2019	TB	GeneXpert MTB/RIF	POC / Motorized tricycle scenario (20km/h)	51 additional healthcare facilities based on geographical models and remote sensing: at least one TB facility in 10 km	Distance/Travel time to the nearest POC facility providing TB testing services. [Cost Distance]	Km/h
2	Database search	Health service providers in Somalia: their readiness to provide malaria case-management	Noor et al. (2009)	Malaria Journal	Retrospective of qualitative survey	Somalia	Puntland, Somaliland, and south-central region of Somalia	3 districts	2007-2009	Malaria	RDT / Microscopy	Hospital – Mother and child Health – Health post / No referral	-	Mean distance from settlement outside the capitals of each district to the nearest service provider	Km
3	Database search	Use of geographically weighted Poisson regression to examine the effect of distance on TB incidence: A case study in Nam Dinh, Vietnam	Viet Bui et al. (2018)	PLoS One	Retrospective	Vietnam	Nam Dinh	229 communes	2012-2015	TB	Smear sputum microscopy – tuberculin skin test – chest X-ray	Provincial hospital (1) – District hospital (12) / No referral	-	GLM et GWPR: Density, poverty, domain, distance – Distance is based on Euclidean distance in Km.	Km
4	Database search	Barriers to completing TB diagnosis in Yemen: Services should respond to patient's needs	Anderson de Cuevas et al. (2014)	PLoS One	Cross-sectional survey and in-depth interviews	Yemen	Sana'a	-	2009 - 2010	TB	-	National Tuberculosis Institute: referral center for TB diagnostics	-	-	-
5	Database search	Scaling up infant diagnosis HIV in Rwanda, 2008-	Binagwaho and al. (2013)	Journal of Public Health Policy	Retrospective	Rwanda	-	-	2008-2010	HIV-exposed infants	Dried Blood Sample by PCR	PMTCT sites: Prevention of mother to child	TRACNet server to send PCR results automatically to mobile phone +	-	-

												transmission / Pickup	PMTCT + 5-day training		
6	Database search	The Current and Future Use of Telemedicine in Infectious Diseases Practice	Coombes et al. (2019)	Current infectious disease report	Review	High Income Countries (HIC)	-	-	Unclear	Hepatitis C, HIV, STIs, TB	-	-	Telemedicine: saving time, reducing travel and missed workdays	-	-
7	Database search	Designing an optimized diagnostic network to improve access to TB diagnosis and treatment in Lesotho	Albert and al. (2020)	PLoS One	Retrospective	**Lesotho**	-	-	2017-2018	TB	GeneXpert	Primary, secondary, and tertiary health tiers / Riders for Health; Courier Company	The design network that gave the lowest transportation cost at each demand level	Travel distance	Meters
8	Database search	Geospatial science and Point-of-Care testing: Creating solutions for population access, emergencies, outbreaks, and disasters	Kost (2019)	Frontiers in Public Health	Review	Multiple	-	-	-	Emergencies, outbreaks, and disasters	-	Point of Care	Time efficiency, POC testing	-	-
9	Database search	Geographic accessibility, readiness, and barriers of health facilities to offer tuberculosis services in East Gojjam Zone, Ethiopia: A convergent parallel design	Andulem et al. (2020)	Research and Reports in Tropical Medicine	Retrospective	Ethiopia	East Gojjam Zone	Kebele (communes)	2018	TB	Microscopy and GeneXpert	Primary (health centers and posts), Secondary (general hospital), Tertiary (specialized hospital) / No referral	-	Euclidean distance (near tool ArcGIS)	Km
10	Database search	"Even though I am alone, I feel that we are many" – An appreciative inquiry study of asynchronous, provider to provider teleconsultations in Turkana, Kenya	Fry et al. (2020)	PLoS One	Retrospective	Kenya	Turkana	County	2017-2018	Several	-	Non-physician clinician (primary healthcare), general physician (specialists, higher level)	Efficiency of referral, equity in access, patient confidence and care seeking behavior, provider motivation and communication	-	-

11	Database search	A web-based system for mapping laboratory networks: Analysis of GLaDMap Application	Mukhi et al. (2012)	Online journal of public health informatics	Retrospective	-	-	-	2011	Information on laboratories production	-	-	*Be used to interact with laboratories around the globe to build capacity and strengthen response*	-	-
12	Database search	Female Genital Tuberculosis in Pakistan – A Retrospective Review of 10-Year Laboratory Data and Analysis of 32 Cases	Fatima et al. (2021)	International journal of Mycobacteriology	Retrospective review	Pakistan	-	289 locations	2007-2016	Female genital TB	Culture or histopathological diagnosis	-	*Xpert Ultra may be a useful tool in improving the yield of molecular diagnosis.*	-	-
13	Database Search	A strategy to increase Tuberculosis case finding in Myanmar's high – risk urban slums	Thu et al. (2015)	Journal of Health Research	Retrospective	Myanmar	-	-	2011-2013	TB	Sputum Smear and Chest X-ray	Motorbikes and public transport (free charges)	*IPC and Pharmacy education and screening: referred when doubts.*	-	-
14	Database search	Temporal trends in correlates of HIV testing uptake in South Africa: evaluation and population-level impacts of socio-economic factors and information sources	Wand et al. (2020)	Journal of Public Health: From theory to practice	Retrospective	South Africa	-	-	2002-2012	HIV	-	-	-	-	-
15	Database search	Performance and user acceptance of the Bhutan febrile and malaria information system: report from a pilot study	Tobgay et al. (2016)	Malaria Journal	Retrospective	Bhutan	Sarpang district	6 remote sites	2013	Malaria	Microscopy	Multiple tier	*Reduced turnaround time, by using mobile phone app.*	-	-
16	Database search	Evaluation of Nepal's Free Health Care	Adhikari et al. (2018)	Journal Nepal Health	Retrospective	Nepal	7 districts	-	Unclear	All	-	Government health facilities	*Free healthcare scheme (FHCS)*	-	-

		Scheme from Health System Perspective: A Qualitative Analysis		Research Council											
17	Snowball search	Optimizing viral load testing access for the last mile: Geospatial cost model for POC instrument placement	Girdwood et al. (2019)	PLoS One	Retrospective	Zambia	-	337 POC candidate on 675 baseline facilities	2020	Viral Load	GeneXpert Omni molecular device	Viral load POC testing device or by linking patient population to a reliable and frequent transport service to centralized laboratories	*POC, POC hubs or Sample transport network – Estimated the cost of each scenario. Optimization of instrument placement by maximizing access and instrument utilization.*	Travel time (>2hours); as a first analysis, to determine the unreached facilities by STN. (salesman problem)	Km/h
18	Snowball search	Sample transport optimization: Mali Pilot Study	Kassambara et al. (2020)	Health Security	Retrospective / Comparative study	Mali	Sikasso region	3 health districts	2016-2017	Several pathogens: meningitis, measles, yellow fever, polio	-	Public transport and postal system to deliver sample from district to DNS	*New partnership for integrated specimen transport: reduced delivered time*	Mean transit time	hours
19	Snowball search	Impact of a borderless sample transport network for scaling up viral load monitoring: results of a geospatial optimization model for Zambia	Nichols et al. (2018)	Journal of the International AIDS society	Retrospective	Zambia		1484 HIV treatment facilities, 26 transport hubs, 19 centralized laboratories	2016-2018	Viral load (HIV)	The Roche Cobas…	Facilities to laboratories	*Cost comparing district-bounded scenario and borderless scenario: Optimized Sample Transport Network*	ArcGIS network analysis tool: samples volumes, distance, and drive times (salesman problem)	Km/h
20	Snowball search	Creating a National specimen referral system in Guinea: Lessons from initial development and implementation	Standley et al. (2019)	Frontiers in Public Health	Retrospective	Guinea	3 prefectures	-	2014-2016	Multiple diseases	-	Motorbikes between health centers and DPS, vehicles between DPS and national level	*Developing a national specimen referral system: creation of hub and spokes, based on questionary*	-	-
21	Snowball search	Building and sustaining	Alemnji et al. (2020)	Journal of Acquired	Literature review	-	-	-	2019-2020	HIV viral load and	-	-	*Optimized diagnostic*	-	-

		optimized diagnostic networks to scale up HIV viral load and early infant diagnosis		Immune Deficiency Syndromes						early infant diagnosis			*networks helping assessing hub and spokes models: reduce time, reduce cost, increase efficiency*		
22	Snowball search	Combatting global infectious diseases: A network effect of specimen referral systems	Fonjungo et al. (2017)	Clinical infectious diseases	Review	-	-	-	-	Infectious diseases	-	Uganda and Ethiopia examples (already read)	*Comparing centralized and decentralized models*	-	-
23	Snowball search	Improved specimen-referral system and increased access to quality laboratory services in Ethiopia: the role of public-private partnership	Kebede et al. (2016)	The Journal of Infectious Diseases	Retrospec tive	Ethiopia	2 regions	-	2008-2012	ART	-	554 laboratories referred specimens to 160 testing laboratories for ART monitoring	*Public-private partnership: improvement of SRN; reduced turnaround time*	-	-
24	Snowball search	Finding the missing patients with TB: Lessons learned from patient-pathway analyses in 5 countries	Hanson et al. (2017)	The journal of infectious diseases	Review	5 countries	-	-	-	TB	GeneXpert, microscopy	Level 0 community care based; level 1 primary healthcare; level 2 ; level 3	*L2 facilities are ideal hub for hub and spokes laboratory model*	-	-
25	Snowball search	Preparing national tiered laboratory systems and networks to advance diagnostics…	Ondoa et al. (2020)	African Journal of Laboratory Medicine	Review	Several	-	-	-	HIV/TB	-	-	-	-	-
26	Snowball search	Bringing Data Analytics to the design of optimized diagnostic networks in LMIC: Process, terms and definitions	Nichols et al. (2021)	Diagnostics MDPI	Review	Several	-	-	-	Several	-	Specimen referral system – national tiered laboratory network	*Diagnostic network optimization: diagnostics should be available, accessible, have a good turnaround time*	-	-

													and a goof quality		
27	Snowball search	Using a GIS to enhance patient access to POC diagnostics in a limited-resource setting	Ferguson et al. (2016)	International journal of health geographics	Retrospective	**Thailand**	Isaan	3 provinces	2010	Heart disease	-	Travel from a place of origin to location of diagnostic and then from diagnostic to care location	*Best POC location using Location-Allocation Tool, follow different implementation strategies: closest road: Network Analysis from ArcGIS*	Travel Time	hours
28	Snowball search	Improving the accessibility and efficiency of POC diagnostics services in LMICs: lean and agile supply chain management	Kuupiel et al. (2017)	Diagnostics MDPI	Review	LMICs	-	-	-	-	-	POC diagnostic should take into consideration the geographical location in which test kits will be used	-	-	-
29	Manuel search	An Integrated Tiered Service Delivery Model (ITSDM) Based on Local CD4 Testing Demands Can Improve TurnAround Times and Save Costs whilst Ensuring Accessible and Scalable CD4 Services across a National Programme	Glencross et al. (2014)	PLoS One	Retrospective	South Africa	9 provinces	52 districts	2009-2012	HIV, ART	CD4 testing	6 hierarchical tiered levels	*The ITSDM offers improved local TAT by extending CD4 services into rural/remote areas with new Tier-3 or Tier-2/POC-Hub services installed in existing community laboratories, most with developed infrastructure*	Euclidian Distance radius (100 km)	Km
30	Manuel search	Estimating Implementation and Operational Costs of an Integrated Tiered CD4 Service including Laboratory and Point of Care Testing in a	Cassim et al. (2014)	PLoS One	Retrospective	South Africa	1 district	Pixley-ka-Seme	2012-2013	HIV, ART	CD4 testing	6 hierarchical tiered levels	*Costs*	-	-

		Remote Health District in South Africa													
31	Database search 2nd	Geographical concentration of falciparum malaria treated in the UK and delay to treatment with artesunate in severe cases: an observational study	Broderick et al (2012)	BMJ	Observational study	UK	-	Hospital level	2008 - 2010	Falciparum Malaria	-	Hub and Spoke system which create delay-to-treatment	-	Diagnosis to treatment time: 1h for Hospital Tropical Disease. 7.5h for artesunate to be couriered. 25h for patient to be transferred	hours
32	Database search 2nd	Dilated eye examination screening guideline compliance among patients with diabetes without a diabetic retinopathy diagnosis: the role of geographic access	Lee et al. (2014)	BMJ Open Diabetes Research and Care	Retrospective study	US	Miami	-	2007-2010	Diabetes	Dilated eye examination	Public transport to eye care facilities	-	Logistic regression: average distance (Euclidean distance) and transit score for compliance	miles
33	Database search 2nd	A mathematical model for designing networks of C-Reactive Protein point of care testing	Lamas-Fernandez et al. (2019)	PLoS ONE	Retrospective study	UK	Southampton Oxford, Isle of Wight, and Lincolnshire	-	unclear	Lower respiratory tract infections	C-Reactive Protein	Walking – travel burden: difference between based (home- GP) and extended travel (Home-GP-POC test) routes	*Mathematical facility location-allocation model, minimizing investment and travel burden for patient*	Open-Source Routing Machine (OSRM) - distance	meters
34	Database search 2nd	Assessing trends in hospital system structures from 2008 to 2015	Walker et al. (2018)	Medical care	Retrospective study	US	-	-	2008-2015	-	-	-	-	Average distance between two pair of hospitals within a given system within each year	miles

35	Database search 2nd	Equity, discrimination, and remote policy: Investigating the centralization of remote service delivery in the Northern Territory	Markham et al. (2015)	Applied geography	Retrospective study	Australia	The Northern Territory	-	unclear	-	-	Road network, and public air transit and ferry services for island population.	*A location-allocation analysis was undertaken to identify this optimal system of reallocated service hubs*	Travel time: estimated as a continuous surface across the state for cartographic purposes using a travel time of 60km/h off the road network	hours
36	Database search 2nd	Internet based consultations to transfer knowledge for patients requiring specialized care: retrospective case review	Kedar et al. (2003)	BMJ	Retrospective study	US	-	-	unclear	Oncology related services	-	-	-	-	-
37	Manuel search	Specimen Referral Network to Rapidly Scale-Up CD4 Testing: The Hub and Spoke Model for Haiti	Frantz et al. (2015)	PubMed	Retrospective study	Haiti	-	-	2011-2014	HIV	CD4 testing	Fourteen hubs serving a total of 67 healthcare facilities have been launched – Hub and Spoke model	*Designed and implemented a national specimen referral network to rapidly scale up CD4 testing*	-	-
38	Manuel search	Uganda's new national laboratory sample transport system: a successful model for improving access to diagnostic services for early infant HIV diagnosis and other programs	Kiyaga et al. (2013)	PLoS One	Retrospective study	Uganda	-	-	2012	Early HIV Infant	Dried blood spots	Evaluate a novel specimen transport network	*Reduce cost transportation and turnaround time with SMS printers*	-	-
39	Manuel search	Sample Transport Optimization: Mali Pilot Study	Kassambara et al. (2020)	Health Security	Retrospective study	Mali	Bamako	3 districts	2016-2017	Meningitis, measles, yellow fever, and polio	-	Specimen transport system, from district to central level.	*Evaluate the effectiveness (ie, timeline, quality of specimen, and cost) of using the trained postal*	-	-

													service for sample transportation.		
40	Advised	Developing strategies for onchocerciasis elimination mapping and surveillance through the diagnostic network optimization approach	Albert and al. (2021)	Frontiers in tropical diseases	Retrospec tive study	DRC and Angola	Bandundu (DRC) and Uige (Angola)	Kwilu, Kwango and Mayi Ndombe provinces (DRC)	-	Neglected tropical diseases – Onchocercia sis	Enzyme-linked immunosor bent assay (ELISA) or rapid diagnostic test (RDT)	Community to health center; community to general hospital; health center to general hospital	*ESS (environmental suitability scores) + scenarios*	Distance Supply Chain Guru	meters
